# Comparison of pEGASUS-HPC and CREDO Heal stent systems deployed via NeuroSpeed percutaneous angioplasty balloon catheter for treatment of acute symptomatic intracranial stenosis and occlusion

**DOI:** 10.1007/s00062-025-01544-1

**Published:** 2025-08-08

**Authors:** Abdallah Aburub, Mohammad Almohammad, Ali Khanafer, Zakarya Ali, Mariana Gurschi, Yashar Aghazadeh, Mete Dadak, Tawfik Moher Alsady, Lars Timmermann, Ole Simon, Anja Gerstner, Bayan Alhaj Moustafa, Oussama Dob, Christopher Nimsky, Benjamin Saß, Hans Henkes, André Kemmling

**Affiliations:** 1 Department of Neuroradiology, Philipps-University Marburg, University Hospital of Giessen and Marburg, Campus Marburg, Marburg, Germany; 2 Neuroradiology, Klinikum Stuttgart Katharinenhospital, Stuttgart, Germany; 3 Central Institute for Diagnostic and Interventional Radiology, Neuroradiology, and Nuclear Medicine, Sana Klinikum Offenbach, Offenbach, Germany; 4 Department of Radiology, St. Vincenz Hospital Paderborn, Paderborn, Germany; 5 Department of interventional Radiology and Neuroradiology, Rhön-Klinikum, Campus Bad Neustadt, Bad Neustadt an der Saale, Germany; 6 Department of Neurology, University Hospital Marburg, Marburg, Germany; 7 Department of Neurosurgery, University Hospital Marburg, Marburg, Germany

**Keywords:** CREDO heal stent, pEGASUS-HPC, Intracranial artery stenosis, Percutaneous transluminal angioplasty, Vessel Occlusion

## Abstract

**Purpose:**

To compare the efficacy and safety of the pEGASUS-HPC and CREDO heal coated stent systems used with the NeuroSpeed percutaneous angioplasty (PTA) balloon catheter, for treating acute symptomatic intracranial artery stenosis (ICAS) with or without acute vessel occlusion (VO).

**Methods:**

This retrospective, multicenter study included patients with ICAS between June-2021 and June-2024 treated with the NeuroSpeed PTA balloon catheter and either stent system. Clinical endpoints included National Institutes of Health Stroke Scale (NIHSS) scores and modified ranking scores (mRS), safety and efficacy endpoints included in-stent thrombosis and in-stent stenosis.

**Results:**

Sixty-nine patients were included (pEGASUS-HPC, *n* = 34; CREDO heal, *n* = 35). Both groups showed significant improvement in arterial diameter post-intervention (pEGASUS-HPC: 0.47 mm (SD 0.27 mm) to 1.69 mm (SD 0.55 mm); CREDO heal: 0.57 mm (SD 0.41 mm) to 1.89 mm (SD 0.62 mm)). Revascularization success, defined as residual stenosis < 50%, was achieved in 32/34 (93.9%) patients in the pEGASUS-HPC group and 33/35 (94.3%) in the CREDO heal group. In-stent re-stenosis occurred in 5/34 (14.7%) of patients in the pEGASUS-HPC group and 2/35 (5.7%) in the CREDO heal group and retreatment with PTA was performed in 3/34 (8.8%) and 2/35 (5.7%), respectively. Peri- or postprocedural in-stent thrombosis occurred in 3/34 (8.8%) of patients in the pEGASUS-HPC group and 2/35 (5.7%) in the CREDO heal group. At 3–6 months, the proportion of patients achieving an mRS score of 0–2 was 25/34 (73.5%) in the pEGASUS-HPC group and 28/35 (80.0%) in the CREDO heal group (*p* = 0.578).

**Conclusion:**

Both stent systems proved effective and safe, showing significant post-intervention arterial dilation, high revascularization rates, and similar functional outcomes (mRS 0–2 at 3–6 months), though they differed in in-stent restenosis rates.

## Introduction

Intracranial artery stenosis (ICAS) is a significant risk factor for recurrent ischemic stroke, with incidence rates ranging from 1 to 50% across various populations [1, 2]. The primary approach for managing ICAS is medical treatment. However, if medical management proves ineffective, percutaneous angioplasty (PTAS) and stenting serves as a highly effective alternative for revascularization [3, 4].

The Wingspan stent, approved by the Food and Drug Administration (FDA) in 2004 for severe medically refractory ICAS, involves navigating the stenosis with a guidewire and deploying a self-expanding stent [5]. However, the SAMMPRIS trial demonstrated that aggressive medical therapy was superior to endovascular treatment with the Wingspan stent for patients with severe ICAS in terms of technical safety and clinical outcomes. These findings led to a paradigm shift in the treatment of ICAS, with a stronger emphasis on dual antiplatelet therapy (APT) using aspirin and clopidogrel for the first 90 days after symptom onset [6].

Following SAMMPRIS, additional trials aimed to refine endovascular therapy. The VAST (Vertebral Artery Stenting Trial) and VISSIT (Vitesse Intracranial Stent Study for Ischemic Stroke Therapy) trials evaluated newer stenting technologies but similarly failed to demonstrate any advantage of stenting over medical therapy [7, 8]. These studies, while utilizing different stent designs and procedural techniques, revealed persistently high periprocedural complication rates, reinforcing the limitations of PTAS as a first-line treatment for ICAS.

Nearly a decade later, the CASSIS trial (China Angioplasty and Stenting for Symptomatic Intracranial Severe Stenosis) reassessed the role of stenting in a more selective population with lower procedural risk. However, the results once again failed to show any clear benefit of stenting over aggressive medical management [9]. Collectively, these Randomized Controlled Trials, including SAMMPRIS, VAST and VISSIT, have contributed to the prevailing view that intracranial stenting should be reserved as a last resort for patients with ICAS who are refractory to medical treatment.

To address the limitations of previous approaches, new PTA balloon catheters, such as the NeuroSpeed, have been developed to simplify stent delivery through a direct one-pass technique [10, 11]. The NeuroSpeed PTA balloon catheter (0.0165’’) can navigate stenotic lesions for both angioplasty and delivery and placement of low-profile self-expanding laser-cut stents including CREDO and pEGASUS-HPC. This system eliminates the need for an exchange maneuver and the associated use of a long exchange wire, thereby reducing risks of wire manipulation and device passage contact with the lesion [10–12].

The CREDO heal stent is a low-profile, self-expanding, closed-cell, laser-cut stent that incorporates surface modification by HEAL technology, an anti-thrombogenic fibrin-heparin coating designed to reduce platelet adhesion [13]. This stent is intended for controlled treatment of ICAS and has shown promising early clinical results, with a significant reduction in stenosis and minimal device-related complications [14]. While approved for use since 2014 [15], its safety and outcomes have not been widely compared with the FDA-approved Wingspan stent system for severe medically refractory ICAS. However, some studies suggest comparable outcomes; for instance, Lin et al. found that the Credo and Wingspan stent systems show similar clinical results, safety profiles, and technical success rates, with no instances of recurrent stroke or mortality observed within one month post-procedure or during the one-year follow-up period [15].

On the other hand, the pEGASUS-HPC stent (Phenox GmbH, Bochum, Germany) is a low-profile, self-expanding, open-cell, laser-cut stent designed for the treatment of intracranial aneurysms and stenosis. Its unique hydrophilic polymer coating (HPC) significantly reduces thrombogenicity, enhancing patient safety [16]. Indicated for both acute ischemic stroke and elective cases [17], the pEGASUS-HPC stent demonstrates high technical success and feasibility, achieving a stenosis reduction of approximately 53% [17]. This stent system was used in a single-center case series, which included 41 patients with 42 stenoses including elective and emergency cases with ICAS [17]. The study reported a 16.3% rate of procedural complications, with a higher occurrence in emergency cases compared to elective ones. Despite these complications, the overall outcomes were favorable, with 62.5% of the stents remaining patent at follow-up imaging. This initial experience highlights the pEGASUS-HPC stent’s efficacy and safety, particularly in emergency situations where traditional thrombectomy has failed.

Both CREDO heal and pEGASUS-HPC stents exhibit a low-profile design, using smaller delivery catheters compared to Wingspan used in the SAMMPRIS trial (0.027 inches vs. 0.0165 inches) [16]. These advancements in stent design and delivery systems have enabled less invasive procedures using smaller access vessels, thereby enhancing the safety and efficacy profiles of newer stents [15, 17]. While individual studies have shown promising results for both stent systems, there remains a significant lack of direct comparative analyses between these two advanced low-profile, coated stent technologies, particularly regarding standardized procedural techniques that utilize the same PTA balloon and antiplatelet regimen. Understanding their relative technical success, safety, and clinical outcomes is of high interest with regard to treatment strategies and improving patient care. Therefore, this study aimed to evaluate and compare the procedural efficacy and safety as well as clinical outcomes of the pEGASUS-HPC stent versus the CREDO heal stent, both exclusively used in conjunction with only the NeuroSpeed PTA balloon catheter for the treatment of acute symptomatic ICAS defined as acute symptomatic high-grade stenosis of > 70% with or without acute vessel occlusion.

## Methods

### Study Design

This retrospective multicenter study was conducted at four neurovascular centers. Data registries were consecutively screened spanning from July 13, 2021, to June 1, 2024. Ethical approval for the study was provided by our local Ethics Committee (IRB: 24-180 RS), which also waived the requirement for informed consent due to the anonymized nature of data collection and analysis. Patients were retrospectively identified from the neurovascular center’s database, ensuring confidentiality through anonymized data collection and analysis.

### Study Participants

Patients were screened according to the following inclusions criteria:

1. Acute neurological deficit due to ICAS or acute vessel occlusion with underlying ICAS.
2. Endovascular treatment by PTA using only the NeuroSpeed balloon catheter followed by release of either the pEGASUS-HPC or CREDO heal stent for ICAS.
3. Multimodal CT imaging upon admission including cerebral computed tomography (CCT), CT angiography (CTA) and CT perfusion with (a) critical perfusion deficit (Tmax > 6 s) involving at least 50% of the involved arterial territory but (b) limited extent of definite early infarct hypodensity defined by Alberta Stroke Program Early CT Score (ASPECTS) 7–10 (or extend less than 20% or perfusion deficit).

### Endovascular Procedures

**Fig. 1:**
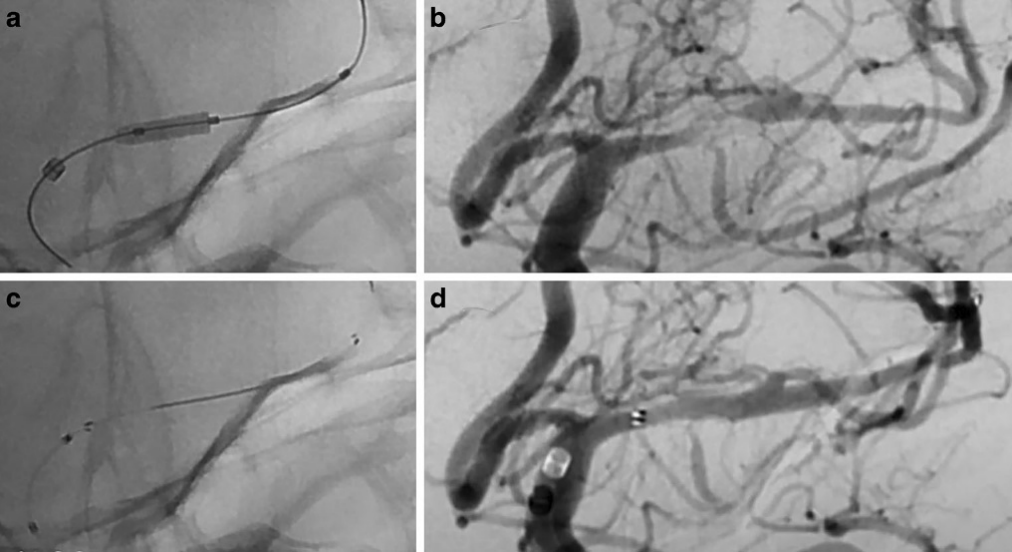
Imaging findings with acute ICAS in the M1 segment of the left middle cerebral artery. (**a** the high-grade stenosis is treated by PTA using the Neurospeed ballon catheter (2.0 mm diameter at 6 bar, length 8.0 mm), **b** After PTA, a Credo heal stent (3.0 × 25 mm is released through the ballon catheter in place (distal and proximal stent markers shown with radiopaque center-strip of pusher wire)), **c** The stenotic lesion of the M1 segment is significantly reduced in (**d**) showing postprocedural revascularization success defined by a residual stenosis grade of < 50%.)

Endovascular procedures were standardized across all participating centers. In cases of vessel occlusion, thrombectomy was initially performed using aspiration and/or a stent retriever, followed by PTA of the remaining stenosis and subsequent stent implantation using the NeuroSpeed PTA balloon catheter. In patients with ICAS without vessel occlusion, only PTA and stenting were performed. Patients were treated exclusively with either the pEGASUS-HPC or CREDO heal stent, based on the attending physician’s discretion, without predefined selection criteria at each participating center (Fig. 1).

The choice between the pEGASUS-HPC and CREDO heal stent was at the discretion of the attending physician, based on the individual clinical scenario and the physician’s familiarity with the devices. No formal or predefined selection criteria were applied. In some cases, local device availability at the participating centers may also have impacted stent selection.

Both stents are compatible with 0.0165″ microcatheters and were implanted using the NeuroSpeed PTA balloon catheter.

### Antithrombogenic Regimen and Follow-up Imaging:

ICAS without vessel occlusion (VO) included treatment-refractory patients on dual APT with either 100 mg acetylsalicylic acid (ASA) and 75 mg clopidogrel daily or 90 mg ticagrelor twice daily. Effective platelet inhibition was tested in all patients (VerifyNow or Multiplate [18]).

ICAS with VO included patients who were initially treated with thrombectomy. The subsequent treatment of ICAS was managed according to a standardized protocol: patients received 500 mg of intravenous ASA prior to PTA and stent implantation. Following PTA and stent implantation, after ruling out possible complications, intravenous tirofiban was administered as a bolus with continued tirofiban infusion (weight-adapted dose). Additionally, 4000 IU of intravenous heparin was administered unless thrombolysis had been performed prior to endovascular therapy. Immediate post-procedure imaging included CCT to rule out hemorrhage or malignant infarction. An oral loading dose of ticagrelor (180 mg) was then administered, accompanied by continued tirofiban infusion for a total of 6 h.

In both ICAS groups, dual APT was continued after treatment for six months. As standard practice, CCT and CTA were performed 6 h after the intervention to evaluate for complications and assess stent patency. Dual APT was continued the following day, consisting of ticagrelor 90 mg twice daily and ASA 100 mg once daily, and continued for six months. At three- and six-month follow-up, digital subtraction angiography (DSA) and CCT or Magnetic Resonance (MR) imaging were performed to rule out new infarction or re-stenosis. Long-term APT was then maintained with ASA 100 mg daily.

### Data Collection

Anonymized data collection included demographic data (e.g., age and gender), clinical parameters (e.g., baseline National Institutes of Health Stroke Scale (NIHSS), baseline arterial diameter, type and location of lesions), procedural details (e.g., employed catheters, stent diameter, stent length, and antiplatelet protocol), and clinical outcome data (e.g., post-intervention mRS, NIHSS, arterial diameter, retreatment, and complications), which were collected retrospectively and analyzed. Image evaluations were conducted using the department’s Picture Archiving and Communication System (PACS). Quality control procedures included the documentation and analysis of all materials used, procedural complications, and their management.

### Clinical and Imaging Endpoints

Grade of stenosis was defined as follows, using the minimum lumen diameter and the reference diameter of the normal parent vessel, which was calculated as the mean of the proximal and distal diameters adjacent to the stenosis:

$$\begin{aligned}{}&{\%}\,\textit{stenosis}=\\&\left[1-\frac{(\textit{diameter}\,of\,\textit{stenosis}}{\textit{diameter}\,of\,\textit{normal}\,\textit{parent}\,\textit{vessel})}\right]\times 100\end{aligned}$$

Procedural efficacy and safety of the stent systems were assessed by revascularization success. Postprocedural revascularization success of ICAS was defined as a residual stenosis grade of < 50%, consistent with definitions of technical success used in major trials such as SAMMPRIS and WEAVE. In VO with underlying ICAS, successful recanalization of VO was defined by Thrombolysis in Cerebral Infarction (TICI) score of ≥ 2b. Safety endpoints included the incidence of complications on periprocedural or follow-up imaging (hemorrhage, vessel dissection, or occlusion). Clinical endpoints included NIHSS at admission and discharge, mRS at discharge and 6 months follow up, and recurrence of ischemic stroke within the follow-up period. Imaging endpoints of re-stenosis on follow-up were graded on a four-stage scale: no stenosis, ≤ 50% mild stenosis, > 50% severe stenosis, and complete occlusion.

### Statistical Analysis

Data were cleaned and analyzed using Statistical Package for Social Science (SPSS; IBM Inc., Windows version 25). The normality of the analyzed data was evaluated using the Shapiro-Wilk test. Categorical data were reported as frequencies and percentages, while continuous data were described as mean and standard deviation (SD). Non-normally distributed variables were presented with the median and interquartile range (IQR). Normally distributed continuous data were analyzed using the independent or paired Student‑T test. In contrast, non-normally distributed variables were analyzed using the Mann-Whitney U or Wilcoxon rank tests. The Chi-square test or Fisher-Exact test was used to assess the association between categorical variables. The results were reported along with the 95% confidence interval (CI), and a *p*-value of less than 0.05 was considered statistically significant.

## Results

### Demographic and Clinical Characteristics

In the comparison, the pEGASUS-HPC (*n* = 34) and CREDO heal (*n* = 35) groups were similar. The mean age was 69.8 years (SD 12.2) in the pEGASUS-HPC group and 72.2 years (SD 12.5) in the CREDO heal group (*P* = 0.067). Males comprised 67.7% of the pEGASUS-HPC group and 60.0% of the CREDO heal group (*P* = 0.509). Arterial status at admission showed no significant difference between groups, with 41.2% (14/34) in the pEGASUS-HPC group and 40.0% (14/35) in the CREDO heal group having ICAS, while 58.9 and 60.0%, respectively, had ICAS presenting with VO (*P* = 0.921). The anatomic location of stenosis or occlusion varied, showing different frequencies. Regarding anterior versus posterior stenosis or occlusion, 52.9% of the pEGASUS-HPC group had anterior involvement, compared to 57.1% in the CREDO heal group, while posterior involvement was noted in 47.1 and 42.9%, respectively (*P* = 0.726). Admission NIHSS scores did not differ between the groups (*P* = 0.689). The baseline NIHSS for the total cohort was 6 (IQR: 3–11) while the CREDO heal group had 7 (IQR: 4–12), as shown in Table 1.

**Table 1:** Demographic and clinical characteristics

Demographic and clinical characteristics	pEGASUS-HPC (*n* = 34)	CREDO heal (*n* = 35)	*P*-value
Age, Mean (SD), years	69.82 (12.22)	72.17 (12.46)	0.067
Gender (Male)	23 (67.7%)	21 (60.0%)	0.509
ICAS without VO	14 (41.2%)	14 (40.0%)	0.921
ICAS with VO	20 (58.9%)	21 (60%)	1.000
*Lesion location (right and left)*			
ACI	7 (20.6%)	2 (5.7%)	–
M1	8 (23.5%)	11 (31.4%)	
M2	0 (0.0%)	4 (11.4%)	
BA	8 (23.5%)	7 (20.0%)	
V4	6 (17.6%)	5 (14.3%)	
ACA/PCA	5 (14.7%)	6 (17.1%)	
Total cohort baseline NIHSS, Median (IQR)	6 (3–11)	7 (4–12)	0.689
Prior anticoagulant therapy	1/34 (2.9%)	4/35 (11.4%)	0.356
*Prior APT (ICAS without VO)*			
SAPT	5/20 (25.0%)	9/14 (21.0%)	0.128
DAPT	4/14 (28.6)	0/14 (0.0%)	0.098
*Prior APT (ICAS with VO)*			
SAPT	5/20 (25.0%)	9/21 (42.9%)	0.326
DAPT	1/20 (5.0%)	0/21 (0.0%)	0.488

*SD* Standard deviation, *NIHSS* National Institutes of Health Stroke Scale, *ICAS* Intracranial artery stenosis, *VO* Vessel Occlusion, *APT* Antiplatelet Therapy, *SAPT* Single Antiplatelet Therapy, *DAPT* Dual Antiplatelet Therapy, *ACI* internal cerebral artery, *M1* the sphenoidal or horizontal segment of the middle cerebral artery, *M2* the insular segment of the middle cerebral artery, *BA* basilar artery, *V4* the intradural or intracranial segment of the vertebral artery, *ACA* Anterior Cerebral Artery, *PCA* Posterior Cerebral Artery, *IQR* Interquartile Range

### Procedural Characteristics

The time from symptom onset to stent implantation was comparable between the pEGASUS-HPC and CREDO heal groups (*P* = 0.395). Predominant arterial access was transfemoral in both groups, with 94.1% in pEGASUS-HPC and 88.6% in CREDO heal (*P* = 0.414), and transradial with 5.9 and 11.4%, respectively. Thrombectomy was performed in 58.8% of pEGASUS-HPC patients and 60.0% of CREDO heal patients (*P* = 1.000).

In the pEGASUS-HPC group, stent lengths ranged from 15 to 30 mm, with diameters of 3.5 mm (*n* = 26) and 4.5 mm (*n* = 12). Analogously, in the CREDO heal group, stent diameters were 2.0 mm (*n* = 1), 3.5 mm (*n* = 14), 4.0 mm (*n* = 12), and 5.0 mm (*n* = 9), with stent lengths ranging from 20 to 30 mm. These numbers include cases requiring additional stents due to multisegmental or long-segment stenosis. The proximal landing zone vessel diameters measured 2.37 mm (SD 0.77 mm) in the pEGASUS-HPC group and 2.55 mm (SD 0.88 mm) in the CREDO heal group (*P* = 0.467). The distal landing zone diameters were 2.08 mm (SD 0.66 mm) and 2.09 mm (SD 1.01 mm), respectively (*P* = 0.950). Stent overlap occurred in 6.1% of pEGASUS-HPC patients and none in CREDO heal (*P* = 0.012), as shown in Table 2.

**Table 2:** Procedure characteristics

Procedure characteristics	pEGASUS-HPC (*n* = 34)	CREDO heal (*n* = 35)	*P*-value
*Arterial Access*	*–*		*0.414*
Transfemoral	32 (94.1%)	31 (88.6%)	–
Transradial	2 (5.9%)	4 (11.4%)	
Thrombectomy (ICAS with VO)	20/34 (58.8%)	21/35 (60%)	1.000
Thrombolysis (ICAS with VO)	8/20 (40.0%)	7/21 (33.3%)	0.751
*Number of devices*			
1	29 (85.3%)	34 (97%)	–
2	4 (11.8%)	1 (2.9%)	
3	1 (2.9%)	0 (0.0%)	
Device Diameter, Mean (SD), mm	3.82 (0.47)	4.00 (0.69)	0.708*
Device Length, Mean (SD), mm	22.3 (3.94)	21.94 (4.31)	0.942
Proximal Landing Zone Diameter, Mean (SD), mm	2.37 (0.77)	2.55 (0.88)	0.467
Distal Landing Zone Diameter, Mean (SD), mm	2.08 (0.66)	2.09 (1.01)	0.950

*ICAS* Intracranial artery stenosis, *VO* Vessel Occlusion, *SD* Standard deviation

*Mann-Whitney U test

### Clinical and Imaging Endpoints

#### Recanalisation Success of ICAS with VO

Successful recanalization of VO in ICAS was achieved in 18/20 (90.0%) of the pEGASUS-HPC group and 19/21 (90.5%) of the CREDO heal group (*P* = 0.410). In the pEGASUS-HPC group (*n* = 20), 2/20 (10.0%) achieved a TICI score of 2b, 2/20 (10.0%) achieved a TICI score of 2c, and 14/20 (70.0%) achieved a score of 3. In the CREDO heal group (*n* = 21), 0/21 (0.0%) achieved a TICI score of 2b, 1/21 (4.8%) achieved a TICI score of 2c, and 18/21 (85.7%) achieved a score of 3.

#### Revascularization Success of ICAS with or without VO

The arterial diameter showed significant improvement from pre-intervention to post-intervention in both the pEGASUS-HPC and CREDO heal groups, as shown in Table 3. In the pEGASUS-HPC group, the mean arterial diameter increased significantly from 0.47 mm (SD 0.27 mm) pre-intervention to 1.69 mm (SD 0.55 mm) post-intervention (*p* < 0.001). Similarly, the CREDO heal group showed a significant increase in mean arterial diameter from 0.57 mm (SD 0.41 mm) pre-intervention to 1.89 mm (SD 0.62 mm) post-intervention (*p* < 0.001). The increase in mean arterial diameter was not significantly different between the two groups (*p* = 0.435). Successful revascularization of stenosis was achieved in 93.9% of the pEGASUS-HPC group and 94.3% of the CREDO heal group, with no statistically significant difference (*p* = 0.089).

**Table 3:** Clinical and procedural endpoints

Clinical and procedural endpoints	pEGASUS-HPC (*n* = 34)	CREDO heal (*n* = 35)	*P*-value
**ICAS without VO**	***n*** **=** **14/34**	***n*** **=** **14/35**	**–**
NIHSS admission, Median (IQR)	3 (2–5)	4 (3–6)	0.078
NIHSS discharge, Median (IQR)	2 (1–3)	3 (2–4)	0.795
*mRS discharge*			
0–2	9/14 (64.3%)	11/14 (78.6%)	–
3–5	5/14 (35.7%)	3/14 (21.4%)	0.277
6	0/14 (0.0%)	0/14 (0.0%)	–
*mRS at 3–6 months*			
0–2	11/14 (78.6%)	12/14 (85.7%)	–
3–5	4/14 (28.5%)	2/14 (14.2%)	0.651
6	0/14 (0.0%)	0/14 (0.0%)	–
**ICAS with VO**	***n*** **=** **20/34**	***n*** **=** **21/35**	**–**
NIHSS admission, Median (IQR)	11 (8–15)	10 (7–14)	0.271
NIHSS discharge, Median (IQR)	6 (4–8)	7 (5–9)	0.695
*mRS discharge*			
0–2	12/20 (60.0%)	15/21 (71.4%)	–
3–5	5/20 (25.0%)	4/21 (19.0%)	0.520
6	3/20 (15.0%)	2/21 (9.5%)	–
*mRS at 3–6 months*			
0–2	14/20 (70.0%)	16/21 (76.2%)	–
3–5	3/20 (15.0%)	2/21 (9.5%)	0.734
6	3/20 (15.0%)	3/21 (14.3%)	–
**TICI score (ICAS with VO)**			
0/1	2/20 (10%)	2/21 (9.5%)	–
2b	2/20 (10%)	0/21 (0.0%)	0.410
2c/3	16/20 (80.0%)	19/21 (90.5%)	–
**Change in arterial diameter**			
Pre-intervention, Mean (SD), mm	0.47 (0.27)	0.57 (0.41)	–
Post-intervention, Mean (SD), mm	1.69 (0.55)	1.89 (0.62)	
**Change in Arterial Stenosis**			
Pre-intervention, Mean % (SD), mm	76.5 (12.9)	74.8 (18.4)	–
Residual Stenosis, Mean% (SD), mm	22.4 (18.3)	17.9% (15.9)	
**Revascularization success**	**93.9% (32/34)**	**94.3% (33/35)**	**0.953**
*Re-stenosis at 3–6 months follow-up*	*5 (14.7%)*	*2 (5.7%)*	*0.259*
Mild	2 (5.9%)	2 (5.7%)	1.000
Severe	3 (8.8%)	0 (0.0%)	0.114
**PTA performed due to re-stenosis**	**3/34 (8.8%)**	**2/35 (5.7%)**	**0.428**
*Periprocedural complications*	*–*		*0.747*
In-Stent Thrombosis	3 (8.8%)	2 (5.7%)	–
Hemorrhage	0 (0.0%)	1 (2.9%)	

*mRS* Modified Rankin Scale, *NIHSS* National Institutes of Health Stroke Scale, *ICAS* Intracranial artery stenosis, *VO* Vessel Occlusion, *SD* Standard deviation, *TICI* Thrombolysis in cerebral infarction, *PTA* Percutaneous Transluminal Angioplasty

#### NIHSS Score

Follow-up was possible for 63 out of 69 patients. The NIHSS score was assessed at admission and discharge in both the pEGASUS-HPC and CREDO heal groups. Patients treated for ICAS without VO had a lower initial NIHSS score than ICAS with VO. In patients with ICAS without VO, the pEGASUS-HPC group had a median NIHSS score of 3 (IQR: 2–5) at admission, which improved to 2 (IQR: 1–3) at discharge. Similarly, the CREDO heal group showed an improvement from 4 (IQR: 3–6) at admission to 3 (IQR: 2–4) at discharge. In patients with ICAS with VO, the pEGASUS-HPC group showed an improvement in NIHSS scores from 11 (IQR: 8–15) at admission to 6 (IQR: 4–8) at discharge, while the CREDO heal group improved from 10 (IQR: 7–14) at admission to 7 (IQR: 5–9) at discharge. The difference in NIHSS scores between the two groups was not statistically significant at admission (*P* = 0.078 for ICAS without VO, *P* = 0.271 for ICAS with VO) and remained non-significant at discharge (*P* = 0.795 for ICAS without VO, *P* = 0.695 for ICAS with VO), as shown in Table 3. Overall, both groups experienced a significant reduction in NIHSS scores following the intervention, with patients with ICAS with VO presenting with higher baseline severity compared to those with ICAS without VO. However, the extent of NIHSS improvement did not significantly differ between the pEGASUS-HPC and CREDO heal groups.

#### mRS Outcomes in Patients with ICAS Without VO

At discharge: In the pEGASUS-HPC group, 64.3% (9/14) of patients achieved an mRS score of 0–2, while 78.6% (11/14) in the CREDO Heal group reached the same outcome. 35.7% (5/14) of pEGASUS-HPC patients and 21.4% (3/14) of CREDO Heal patients had an mRS of 3–5, with no patients having an mRS of 6. At 3–6 months: The proportion of patients with an mRS of 0–2 increased to 78.6% (11/14) in the pEGASUS-HPC group and 85.7% (12/14) in the CREDO Heal group. The proportion with an mRS of 3–5 decreased to 28.5% (4/14) and 14.2% (2/14), respectively.

#### mRS Outcomes in Patients with ICAS with VO

At discharge: 60.0% (12/20) in the pEGASUS-HPC group and 71.4% (15/21) in the CREDO Heal group achieved an mRS score of 0–2. 25.0% (5/20) of pEGASUS-HPC patients and 19.0% (4/21) of CREDO Heal patients had an mRS of 3–5, while 15.0% (3/20) and 9.5% (2/21), respectively, had an mRS of 6. At 3–6 months: The proportion of patients with an mRS of 0–2 increased to 70.0% (14/20) in the pEGASUS-HPC group and 76.2% (16/21) in the CREDO Heal group. Conversely, the proportion of patients with an mRS of 3–5 decreased to 15.0% (3/20) and 9.5% (2/21), respectively, while the proportion of patients with an mRS of 6 remained unchanged at 15.0% (3/20) in the pEGASUS-HPC group and slightly increased from 9.5% (2/21) to 14.3% (3/21) in the CREDO Heal group, as shown in Table 3.

Overall, at 3–6 months, a favorable functional outcome (mRS 0–2) was achieved by 73.5% (25/34) of patients in the pEGASUS-HPC group and 80.0% (28/35) in the CREDO Heal group, including those with and without VO; this difference was not statistically significant (*p* = 0.578).

#### Complications

Complications included in-stent thrombosis in 3 patients (8.8%) in the pEGASUS-HPC group (none with infarcts) and 2 patients (5.7%) in the CREDO heal group (both with infarcts). Post-procedural new infarcts without stent occlusion or stenosis occurred in 2 patients (5.9%) in the pEGASUS-HPC group and 3 patients (8.6%) in the CREDO heal group (*p* = 0.747). Additionally, in the CREDO heal group, one patient (2.9%) experienced severe hemorrhagic complications, including intracerebral hemorrhage (ICH) and subarachnoid hemorrhage (SAH) post-interventionally. Mortality rates at discharge were 8.8% for the pEGASUS-HPC group and 5.7% for the CREDO heal group (*p* = 0.673).

#### Recurrent Stroke, Restenosis and Retreatment

Recurrence of ischemic stroke was noted in 4 patients (12%) in the pEGASUS-HPC group and 2 patients (6%) in the CREDO heal group (*p* = 0.670). Mild stenosis was observed in 5.9% (2/34) of the pEGASUS-HPC group and 5.7% (2/35) of the CREDO heal group, while severe stenosis occurred only in the pEGASUS-HPC group (8.8%, 3/34) (*p* = 0.259). Retreatment was performed in 4 patients (11.8%) from the pEGASUS-HPC group, including 3 PTA procedures (8.8%) and 1 re-thrombectomy (2.9%). In the CREDO heal group, 3 patients (8.6%) underwent retreatment, including 2 PTA procedures (5.7%), 1 (2.9%) re-thrombectomy, with one patient (2.9%) receiving both treatments.

## Discussion

This retrospective study compared the procedural efficacy and safety of the PEGASUS-HPC stent versus the CREDO heal stent, both combined with the NeuroSpeed PTA balloon catheter in the treatment of ICAS. Our findings demonstrated a significant post-intervention increase in arterial diameter in both groups. Revascularization success did not significantly differ between the pEGASUS-HPC group and CREDO heal group (93.9% vs. 94.3%, respectively). These results indicate that both stent systems are effective in achieving significant revascularization, aligning with recent European Stroke Organisation guidelines emphasizing the role of intracranial stenting as a viable option for symptomatic ICAS [19].

To date, numerous studies have assessed various stent technologies for ICAS. However, our study is the first to directly compare two laser-cut stent systems in a patient cohort under standardized procedural conditions. Unlike previous studies, which included heterogeneous procedural techniques and device selections, we ensured that all interventions were performed using the same PTA balloon system (NeuroSpeed) and an identical antiplatelet regimen. This controlled setting minimizes confounding factors and allows a true head-to-head comparison of these two modern low-profile stent technologies in ICAS treatment.

Similar to our findings, a single-center case series study conducted by Pielenz et al. showed that in patients with ICAS who underwent an initial treatment or retreatment of the index lesion, the pEGASUS-HPC stent achieved a 100% revascularization success rate, which is higher than our results. They also showed that the percentage of stenosis was changed from 88% pre-intervention to 35% post-intervention, with a mean percentage of improvement of 53% [17]. This is consistent with other studies indicating the effectiveness of stenting plus medical therapy in reducing stroke risks [8]. Similarly, Dorn et al. reported multicenter data on the CREDO heal stent, involving 81 patients across 12 stroke centers. Their findings demonstrated a final successful recanalization rate (TICI 2b-3) of 95.1%, with only 4.9% experiencing periprocedural complications [13]. Additionally, in the Lin et al. study, the authors showed that the CREDO heal stent resulted in a technical success of 92% compared to 94% in the Wingspan stent (*p* = 1.00) [15]. They defined technical success as the successful deployment of the stent with a residual stenosis of less than 30% [8, 15], a key factor in procedural success [17, 20]. These findings demonstrate that both the pEGASUS-HPC and CREDO heal stent systems are viable options for the treatment of ICAS, with comparable efficacy.

Our findings showed that neurological deficit by NIHSS scores (admission to discharge) improved significantly in both stent groups, with no significant difference between them. Disability by mRS scores (discharge to 6‑month follow-up) improved in both groups, also with no significant differences between groups. These results reinforce previous research highlighting that both stenting and optimal medical therapy contribute to clinical improvement by reducing neurological deficits and disability [21]. On the other hand, Pielenz et al. showed that the improvement in mRS between baseline and discharge post-pEGASUS-HPC was not significant [17]. This could be attributed to several factors, including the non-use of a PTA-balloon catheter and the different inclusion criteria.

The distribution of TICI scores, indicating the degree of reperfusion, showed a high proportion of patients with successful recanalization of VO in ICAS using either stent system with no statistically significant difference (*p* = 0.429). In a study about early results of 17 patients with ICAS who were treated with the CREDO heal stent and the NeuroSpeed PTA balloon catheter, about 82% of the patients achieved a final TICI score of ≥ 2b, with only one device-related complication [22]. In our study, successful recanalization of VO was achieved in 90.0% of the pEGASUS-HPC group and 90.5% of the CREDO heal group (*P* = 0.410), demonstrating the comparable efficacy of both stent systems in achieving effective reperfusion.

Complications such as in-stent stenosis and thrombosis were reported in both groups, with no significant differences. The majority of patients who experienced in-stent stenosis were mild. The incidence of procedural complications, including infarction, was comparable between the two stent systems. Lin et al., showed that the rate of In-stent stenosis was 17% in the CREDO heal group, compared to 24% in the Wingspan group (*p* = 1.00), which is slightly higher than our findings [15]. This could be attributed to the use of a PTA-balloon catheter in our study, which was not the case in their study. Regarding the rate of retreatment, both stent groups were similar (CREDO heal 8.6%, 3/35 vs. pEGASUS-HPC 11.8%, 4/34). The overall safety profile of both stents was comparable (zero mortality at 3 months in both stent groups treated for ICAS without acute VO). This aligns with findings from the WEAVE trial, which demonstrated the importance of proper patient selection and stent choice in optimizing long-term outcomes when treating subacute symptomatic intracranial atherosclerotic disease [23].

The comparable positive outcomes between the pEGASUS-HPC and CREDO heal stent systems suggest that both are effective and safe options for treating ICAS. Future studies should include larger, multicenter, randomized controlled trials to confirm these findings and provide more robust evidence on the comparative efficacy and safety of the pEGASUS-HPC and CREDO heal stent systems (e.g., by using different percutaneous transluminal angioplasty methods and including other stent systems). Additionally, long-term follow-up studies are needed to evaluate the durability of revascularization and the long-term clinical outcomes associated with these stent systems. The importance of long-term follow-up is underscored in the 2021 AHA/ASA stroke prevention guidelines, which recommend continuous assessment of intracranial stenosis patients to identify potential restenosis and recurrent stroke risks [24].

This study has limitations, including its retrospective design, which may have introduced selection bias. The sample size was relatively small, limiting the generalizability of the findings. Additionally, the study relied on retrospective data collection, which may have resulted in incomplete or inaccurate data. Finally, the study population was heterogeneous, consisting of acute cases with vessel occlusion treated in emergency settings and patients with recurrent symptoms treated electively. This heterogeneity could have influenced the results, as treatment approaches and outcomes may differ between these patient groups. Moreover, the follow-up period of 3–6 months may be insufficient to assess long-term vessel patency and stroke recurrence. Restenosis and retreatment rates may increase over time and therefore require extended monitoring in future studies. Future studies should include longer-term follow-up to assess sustained vessel patency, functional outcomes, and the durability of the antirestenotic effect. Prospective, multicenter trials with larger cohorts are warranted to validate these preliminary findings and to establish standardized treatment strategies for ICAS.

In conclusion, this retrospective study found that both the pEGASUS-HPC and CREDO heal stent systems are comparable in the management of patients with ICAS. Both stent systems demonstrated significant improvements in revascularization and clinical outcomes, with comparable rates of complications. These findings support the use of both stent systems in clinical practice, providing valuable insights for clinicians in the management of ICAS. Further research is warranted to validate these results and explore long-term outcomes.

## Data Availability

The data supporting the findings of this study are available from the corresponding author upon reasonable request.
